# The Role of Physisorption and Chemisorption in the Oscillatory Adsorption of Organosilanes on Aluminium Oxide

**DOI:** 10.3390/polym11030410

**Published:** 2019-03-04

**Authors:** Ruby A. Sims, Sarah L. Harmer, Jamie S. Quinton

**Affiliations:** Flinders Institute for Nanoscale Science and Nanotechnology, Flinders Microscopy and Microanalysis, College of Science and Engineering, Flinders University, GPO Box 2100, Adelaide, SA 5001, Australia; ruby.sims@flinders.edu.au (R.A.S.); sarah.harmer@flinders.edu.au (S.L.H.)

**Keywords:** oscillatory adsorption, organosilane, self-assembly, physisorption, chemisorption

## Abstract

The effect of physisorbed and chemisorbed species on the time-dependent self-assembly mechanism of organosilane films has been investigated on aluminium oxide using X-ray Photoelectron Spectroscopy. The role of physisorbed species was determined through their removal using a simple rinsing procedure while monitoring film substrate coverage. Removing physisorbed species from Propyldimethylmethoxysilane films, shown to follow a Langmuir-type adsorption profile, reduces the substrate coverage initially but quickly results in coverages equivalent to films that did not undergo a rinsing procedure. This indicates that all Propyldimethylmethoxysilane molecules are covalently bound to the substrate following 15 s of film growth. Removing physisorbed species from films, which have been shown to follow an oscillatory adsorption profile, Propyltrimethoxysilane and Propylmethyldimethoxysilane, reveal the persistence of these oscillations despite a reduction in silane substrate coverage. These results not only confirm the presence of two thermodynamically favourable phases in the condensation equilibrium reaction as physisorbed and chemisorbed species, but also indicate that the desorption of species during film growth involves both states of chemical binding.

## 1. Introduction

The use of silane molecules as coupling agents and adhesion [1] promoters is extensive and broadly interdisciplinary from medical applications in dentistry [2] to anti-corrosion [3] or anti-microbial [4] coatings. The versatility of these molecules stems from their ability to modify the properties of hydroxylated substrates and provide a new substrate for further functionalisation, with minimal environmental impact [1]. The widespread exploitation of this self-assembly process lends itself to further investigation of the molecular level silane-substrate interactions, in order to intelligently design silane molecules and experimental conditions. While the mechanism of alkoxysilane self-assembly in solution is well known [5], the mechanism of alkoxysilane substrate condensation is not as simple. The presence of an oscillatory behaviour has been reported for silane coatings with more than one hydrolysable group, such as Propyltrimethoxysilane (PTMS) [6,7,8] and Propylmethyldimethoxysilane (PMDMS) [8], as a function of both film growth time and silane concentration in solution. In contrast, restricting the number of hydrolysable groups to one in the case of Propyldimethylmethoxysilane (PDMMS) results in a Langmuir-type adsorption profile on the native oxide of polycrystalline aluminium [8].

The adsorption of molecules to a substrate is often described as physisorption or chemisorption depending on the strength of the interaction between the substrate and adsorbate. Physisorption is a broad term that describes all weak electrostatic interactions including Van Der Waals interactions, dipole-dipole and London forces. The formation of these weak physisorbed interactions between the adsorbate and substrate typically range from 0.2 to 4 kJ/mol [9]. These bonds are considered the weakest of interactions and can be easily broken. Chemisorption occurs when the adsorbate becomes covalently bound to the substrate via the sharing or transfer of electrons, with interactions that are typically two orders of magnitude stronger than that of physisorbed species. The strength of hydrogen-bound interactions lies between that of physisorbed and chemisorbed species at 12–30 kJ/mol [9] but is often described as a physisorbed interaction. The formation of chemisorbed substrate-adsorbate bonds can originate from physisorbed interactions and hydrogen-bound species, making the rate of condensation dependent upon the rates of both physisorption and chemisorption.

As the adsorption of PDMMS follows a Langmuir-type growth mechanism, whereby molecules approach and adsorb onto the substrate until all of the free substrate sites are occupied [10], it has been previously described by a 2-component model of self-assembly, originally proposed by Quinton et al. [11,12]. Component 1, indicated by *θ*_1_ represents the total fraction of species on the substrate that is physisorbed. While the 2-component model identifies *θ*_1_ as hydrogen bound species, any combination of these physisorption mechanisms may be involved. The second component, *θ*_2_, are covalently bound species; which in the case of silane films on aluminium oxide correspond to the presence of Si–O–Al bonds [13]. *θ*_1_ and *θ*_2_ can represent the same silane species on the substrate, with the conversion of *θ*_1_ to *θ*_2_ resulting in an exclusively covalently bound film [12]. Although the formation of a covalent adsorbate-substrate bond is thermodynamically favourable, such a bond is only possible via the formation of a hydrogen bound intermediate species, as proposed by Arkles [14] and is described by the 2-component model.

**Figure 1:**
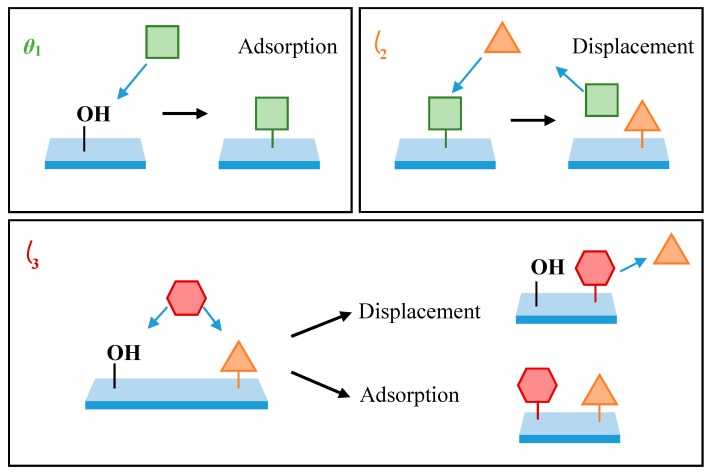
The 3-component model used to describe the oscillatory nature of silane adsorption on metal oxide substrates, adapted from Quinton et al. [11,12]. Silicon containing species *θ*_1_ and *θ*_3_ are represented by squares and hexagons, while the non-silicon containing species *θ*_2_ (H_2_O) is represented by a triangle.

While a simple 2-component model can be used to describe the formation of a PDMMS monolayer, this description requires a molecule which cannot act as a substrate for additional growth. Hence, the model used to explain PDMMS cannot be applied to PTMS or PMDMS. The 3-component model generated by Quinton et al. [11,12] used to describe the oscillatory nature of PTMS adsorption on metal oxide substrates requires 3 unique components assigned *θ*_1_, *θ*_2_ and *θ*_3_ to create a single oscillation in silane surface coverage. In an example of the model shown in Figure 1 below, *θ*_1_ represented by a square and *θ*_3_ represented by a hexagon are described as silane species bound to the substrate, while *θ*_2_ (represented by a triangle) denotes a water molecule. Adsorption of the silane species *θ*_1_ occurs upon exposure to a hydroxylated surface via a condensation reaction. The displacement of *θ*_1_ by *θ*_2_ results in the desorption of silane species from the surface and creates a free adsorption site available for further adsorption of silane species, *θ*_3_. It is speculated that desorption resulting in oscillation is due to a reversal in the dynamic equilibrium condensation reaction in response to an increase in the local water concentration at the film-substrate interface.

The general mechanism reveals how the presence of three components, each with their own adsorption or desorption process and unique rate constant, produce a mathematical function to fit the single oscillation reported experimentally for PTMS [6]. However, contrary to the 2-component model, the 3-component model shown in Figure 1 does not distinguish between physisorbed and covalently-bound adsorption to the substrate.

Studies that over extended silane-substrate exposure times revealed the presence of multiple oscillations in surface coverage, which required the addition of components to the model shown above in order to describe the behavior [8]. While the kinetics of a multilayer system with multiple oscillations in substrate coverage becomes increasingly complex and a 2-component model can no longer be used to represent the kinetics of self-assembly, the thermodynamics requiring the conversion of hydrogen bound to covalently bound species remain the same. To explore this further, the role of physisorbed and covalently bound species on the substrate condensation kinetics during the oscillatory behaviour of film growth is examined.

A curing procedure is often performed on silane films in order to force the dynamic equilibrium condensation reaction to completion; with curing shown to create more siloxane bonds, decrease film thickness and increase film density when compared to uncured films [15]. The density of PTMS on aluminium oxide has been mapped and depth profiled with results indicating the presence of two different film morphologies on the surface of un-rinsed films in the form dense, highly oligomerised islands surrounded by a monolayer-like film [16]. In theory, forced removal of water from the film via curing results in a film containing only covalently bound silane species (*θ*_2_ of the 2-component model). A limitation of both models is that they do not take adsorbate-adsorbate interactions into account and only consider a system where adsorbates interact with the substrate. The 2- and 3-component models describe a net adsorbate-substrate behaviour, however at a molecular level any number of physical interactions can occur. For instance, in the 2-component model hydrogen bound *θ*_1_ can be displaced by another *θ*_1_ silane species prior to its conversion to *θ*_2_. Mathematically, all *θ*_1_ silane species are treated as equivalent based on the final product and thus the model holds. 

In order to determine the role of physisorbed species in the adsorption profiles of organosilanes PTMS, PMDMS and PDMMS, rather than forcing all physisorbed species to become covalently bound (*θ*_1_ converted to *θ*_2_) via curing, physisorbed species (*θ*_1_) could be removed from the substrate prior to their conversation to the covalently bound (*θ*_2_) with a rinsing procedure. Monitoring the stability of the film post cure can also indicate the role of covalently bound species and the stability of films during a rinsing procedure.

## 2. Experimental

Propylmethyldimethoxysilane (PMDMS) 97% and Propyldimethylmethoxysilane (PDMMS) 97% were purchased from Silar Laboratories, Riegelwood, NC USA. Propyltrimethoxysilane (PTMS) of 97% purity, aluminium foil substrates of 0.5mm thickness at 99.99% purity were purchased from Sigma Aldrich, (Castle Hill NSW, Australia). Due to its high purity it could be assumed that there was little variation across the substrate surface, important when creating a homogenous oxide layer. In order to remove and replace the existing oxide layer, creating a reproducible surface for silane condensation, aluminium substrates were abraded with 1200 grade wet-dry sandpaper, rinsed with Milli-Q water and ultrasonicated in a pH3 acetic acid solution at room temperature for one hour. Previous studies have shown this method to result in a reproducible oxide layer [16]. 1% *v*/*v* silane solutions were prepared in a 1:1 H_2_O:EtOH solvent altered to pH 3 with glacial acetic acid and stirred until the silane solution was considered hydrolysed through observing complete dissolution of silane in the aqueous solvent [8]. Silane films were prepared by submerging aluminium substrates in the silane-solvent solution for the prescribed time. Samples which were described as ‘rinsed’ were washed at this point with a steady stream of pH 3 CH_3_COOH (in Milli-Q water) for 5 s to remove physisorbed species. All samples were then dried with N_2_ and cured at 80 ℃ for one hour.

XPS was utilised to determine the average elemental composition and to ascertain a value representing silane substrate coverage. A value of substrate coverage as a function of time was quoted as the ratio of Si:(Si+Al), resulting in a value that tended towards 1 and thus was more sensitive to lower substrate coverage values. Each data point represented the average of three measurements with the error given as the standard deviation. A Leybold-Heraeus LHS-10 X-ray Electron Spectrometer generating Mg K_α_ soft X-rays with an energy of 1253.6 eV [17] using a SPECS XR-50 Dual-Anode X-ray source was used for all measurements. A base pressure of 2.0 × 10^−9^ Torr, take-off angle of 90° and pass energy of 20 eV were set for each sample. CASA XPS version 2.3.15 dev87 ©2009, a licensed peak fitting software designed specifically for the analysis of XPS data was employed for all curve fitting using a mixture of Gaussian and Lorentzian peak shapes, with a Shirley background subtraction [18]. All spectra were normalised to the main C-C *1s* peak component at 285 eV [17,19,20,21]. While Si *2p* peaks are generally fit at 99 eV [17], Si *2p* peaks for silanes were fit at a higher binding energy of 103 eV [22] due to the presence of Si–O bonds indicative of silane molecules. Si *2p* peaks in this report were fit as a single peak component as no further deconvolution was possible. Al *2p* peaks were first fit with a single component representing the Al_2_O_3_ on Al foil at 76 eV, and 2 components at 73 eV representing all aluminium bonds of Al *2p*_1/2_ and Al *2p*_3/2_ [21]. An example of Al *2p* and Si *2p* high resolution spectra can be seen in the Supplementary Materials. 

## 3. Results and Discussion

**Figure 2:**
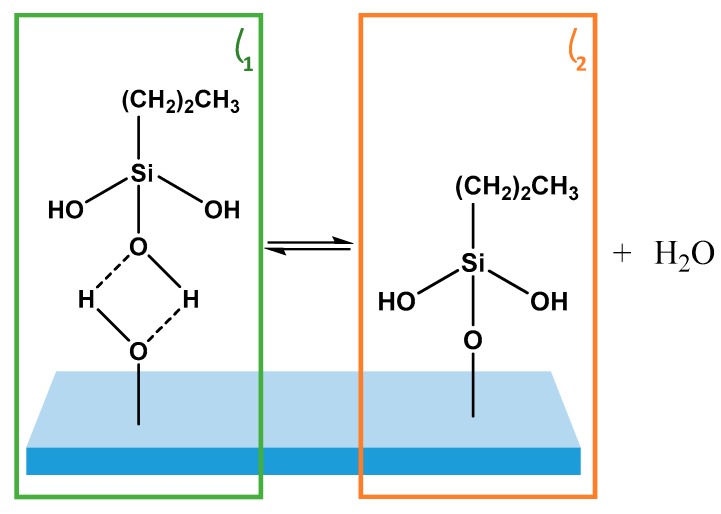
The conversion of hydrogen bound *θ*_1_ represented by a PDMMS monomer (**green**) to *θ*_2_, a covalently bound PDMMS monomer (**orange**) in the 2-component model.

The impact of physisorbed species on the substrate condensation kinetics of the Langmuir-type film growth of PDMMS was investigated by removing physisorbed species *θ*_1_ from the substrate by rinsing the film pre-cure; effectively separating the 2-components, *θ*_1_ and *θ*_2_ shown in Figure 2. A limitation of this method is that it does not discriminate between the types of physisorbed species on the substrate. While the mechanism clearly defines the formation of a hydrogen bound species, any combination of these physisorbed interactions may be present on the substrate, the difference between which cannot be determined by rinsing the film. This does not prevent the role of these species from being determined, however, as it is not the rinsed solution which is examined but rather the film that remains on the substrate. Post rinse it is assumed that silane species which remain on the substrate are covalently bound. In films which are not rinsed, removal of all remaining water from the substrate by curing forces the condensation reaction to completion, effectively converting any hydrogen bound to covalently bound silane species (*θ*_1_ to *θ*_2_). While rinsed films were also cured, physisorbed species had already been removed from the substrate.

**Figure 3:**
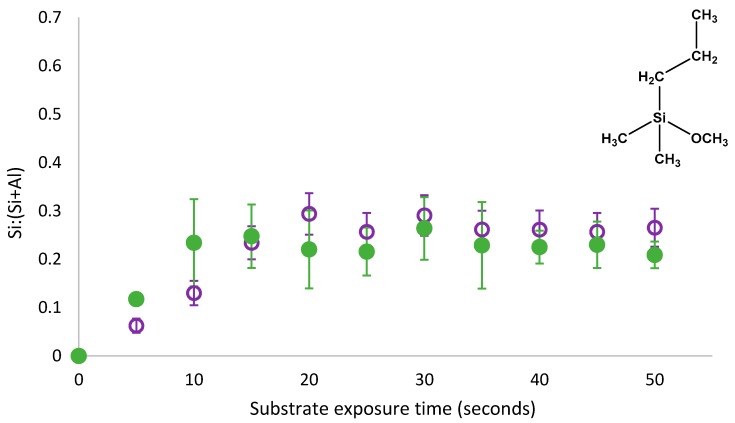
Langmuir-type adsorption of 1% PDMMS in 1:1 EtOH:H_2_O solvent at pH3 on aluminium foil substrates, measured using XPS. Upon removal from solution, samples films were either dried with N_2_ (solid green circles) [8] or rinsed with pH_3_ CH_3_COOH (open purple circles) prior to drying and cured.

The rate of substrate-adsorbate interactions and the formation of silane-metal bonds is dependent on the concentration of metal-OH bonds on the substrate, considering these interactions enable a first order kinetic equation to be used to explain the creation of PDMMS films. As *θ*_1_ and *θ*_2_ represent the same silane molecule on the substrate and the difference is simply whether it is hydrogen- or covalently- bound to the substrate, *θ*_1_ can be removed by rinsing the film prior to its conversion to *θ*_2_. The ability to separate these species gave an insight into the rate of *θ*_1_ (hydrogen bound) to *θ*_2_ (covalently bound) conversion on the substrate. If the original *θ*_1_ and final *θ*_2_ were not a result of a conversion of the same molecule from hydrogen bound to covalently bound species and rather the result of a displacement of *θ*_1_ by another PDMMS molecule creating a new *θ*_1_ which was then effectively converted to *θ*_2_, the same logic could be applied. As the Langmuir type adsorption profile of PDMMS has been reported previously [8], it was directly compared to the PDMMS coverage for rinsed films and is shown in Figure 3.

The removal of physisorbed species from the substrate of PDMMS films revealed a reduction in the amount of silane on the substrate between 0.06 and 0.1 Si:(Si+Al) during the initial stages of film growth. There was no significant difference in the substrate coverage observed for films containing physisorbed species after 15 s of film growth with both adsorption profiles following the same trend and substrate coverage. Direct comparison of films containing both physisorbed and covalently bound (not rinsed) and simply covalently bound species (rinsed films) on the substrate indicated the removal of physisorbed species from the substrate shifting the plateau in substrate coverage and the point where film growth was complete from 10 s to 15 s of film growth. Similar substrate coverages for rinsed and non-rinsed samples indicated that there were very few physisorbed species on the substrate of PDMMS films after 15 s of film growth as the majority of *θ*_1_ was converted to *θ*_2_ after 15 s. For some exposure times shown in Figure 3, rinsed samples were reported to have a higher substrate coverage than non-rinsed samples as the reported values were the average of three replicates, however rinsed and no-rinsed samples fell within the standard deviation error. 

As the kinetics of silane self-assembly were investigated the thermodynamics of the system must also be considered. The conversion of *θ*_1_ to *θ*_2_ as described in the kinetic model is supported by the thermodynamically favourable mechanism of covalent adsorbate-substrate bond formation, which occurs via an intermediate hydrogen bond. The potential energy diagram originally described by Prutton [23] and adapted for silane condensation by Sims et al. [8] describes chemisorption on a planar substrate via the conversion of hydrogen bound to covalently bound adsorption when an equilibrium is forced to completion. While the formation of the covalently bound *θ*_2_ is thermodynamically favourable, it can only occur once the adsorbate is in the hydrogen bound potential well as *θ*_1_, when it can then cross the diffusion energy barrier to become *θ*_2_. The creation of multilayer films from silane molecules with the ability to condense onto both the substrate and another silane molecule, significantly increases the complexity of the self-assembly mechanism and creates the oscillatory behaviour observed for PTMS and PMDMS. Since this multilayer system has now been shown to exhibit multiple adsorption oscillations, the kinetic model required to describe it must be more complex than the models previously reported. However, the thermodynamics of adsorption remains the same with the two distinct states of molecular binding of physisorption and chemisorption. 

As described in Figure 1, in order to create the oscillation in surface coverage reported for organosilane molecules with more than one hydrolysable group, displacement by a non-silicon containing species must occur. It is proposed that this displacement is caused by water molecules that are a by-product of condensation reactions at neighbouring adsorption sites. This local increase in water concentration shifts the dynamic equilibrium in favour of unbound silane species, resulting in desorption. As the amount of silane adsorbed on the substrate was not found to decrease after 15 s of PDMMS self-assembly, it could be said that the rinsing procedure did not contribute to this displacement mechanism as it did not remove covalently bound silanes from the substrate, further supporting the proposed mechanism of silane desorption via water molecule displacement.

While the presence of physisorbed and covalently bound species in the self-assembly of the Langmuir-type growth of PDMMS has been established in Figure 3, the role of these species in the oscillatory growth mechanism reported for PTMS and PDMMS was unknown. If covalently bound species were not involved in the oscillatory mechanism, the removal of physisorbed species form the substrate would reveal an adsorption curve similar to the Langmuir-type adsorption seen in Figure 3. To determine if the oscillation in substrate coverage could be directly related to either physisorbed or covalently bound or if both species were involved in this oscillation, physisorbed PTMS and PMDMS species were removed from the substrate while monitoring the time dependent adsorption behaviour.

**Figure 4:**
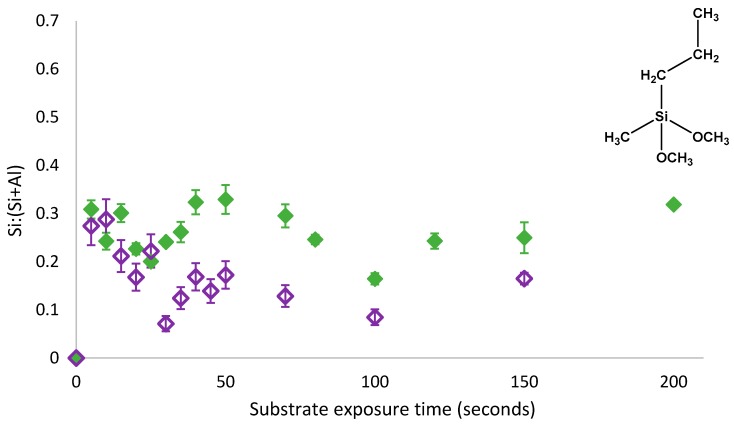
Oscillatory adsorption of 1% PMDMS in 1:1 EtOH:H_2_O solvent at pH3 on aluminium foil measured using XPS. Upon removal from solution, films were either dried with N_2_ (solid purple diamonds) [8] or rinsed with pH3 CH_3_COOH (open purple diamonds) prior to drying and curing.

As shown in Figure 4, an oscillatory adsorption behaviour persisted despite the removal of physisorbed PMDMS species from the surface and thus, this oscillatory behaviour must have been due to the oscillations in both physisorbed and covalently bound species present on the substrate. The presence of physisorbed species did not appear to affect the rate of oscillation, but simply increased the amount of silane present on the substrate between 0.03 and 0.16 Si:(Si+Al). Following the removal of physisorbed species, the substrate coverage trend still followed the 3-component model where components were labelled *θ*_1_, *θ*_2_ and *θ*_3_; indicating that *θ*_1_ and *θ*_3_ in the 3-component model proposed by Quinton et al. [11,12] represented both physisorbed and chemisorbed species on the substrate. As reversal of the condensation reaction was occurring on the species, which was both physisorbed and covalently bound to the substrate, a non-silane component capable of shifting the dynamic condensation equilibrium reaction must have been present in solution; once again indicating that it is water trapped under the film which created an increase in the localised water concentration, shifting the dynamic equilibrium resulting in a reversal of the condensation reaction. To confirm the mechanism of oscillatory growth, the self-assembly of PTMS was also investigated.

**Figure 5:**
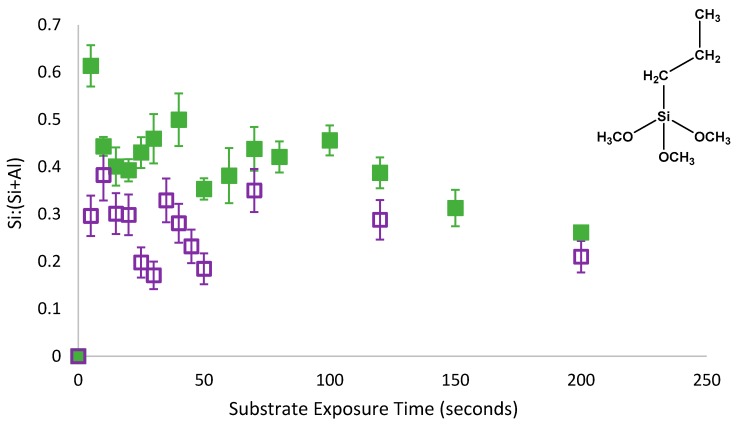
Oscillatory adsorption of 1% PTMS in 1:1 EtOH:H_2_O solvent at pH3 on aluminium foil substrate measured using XPS. Upon removal from solution, films were either dried with N_2_ (solid green squares) [8] or rinsed with pH3 CH_3_COOH (open purple squares) prior to drying and curing.

As with PMDMS, the oscillation of PTMS silane coverage persisted despite the removal of physisorbed species from the substrate (Figure 5). However, contrary to PMDMS, the removal of physisorbed species from the substrate drastically reduced the amount of silane on the substrate after 5 s of film growth; a Si:(Si+Al) of 0.32 for PTMS compared to just 0.03 for PMDMS. This reduction in the initial substrate coverage may indicate that the majority of PTMS molecules were initially physisorbed to the substrate, or that the majority of species physisorbed to the substrate were larger oligomers, having a greater impact on overall substrate coverage upon their removal. To determine the efficacy of the curing process in removing water as well as testing the stability of the film, the surface coverage of a cured PTMS film was examined, films were rinsed post analysis and the silane surface coverage re-measured.

**Figure 6:**
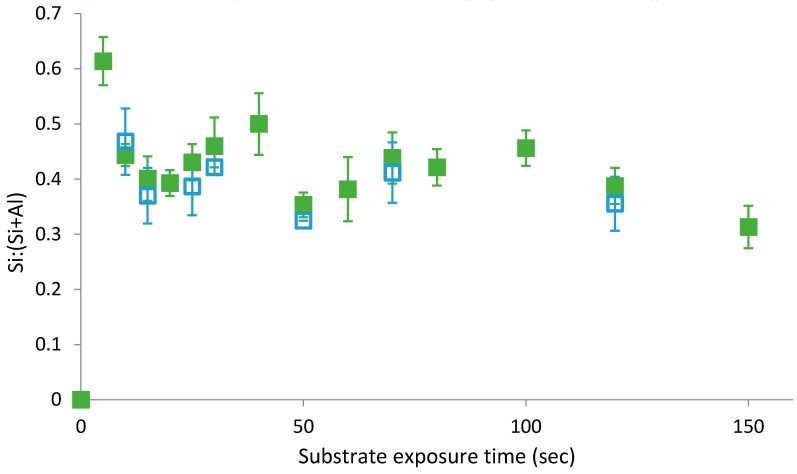
Oscillatory adsorption of 1% PTMS in 1:1 EtOH:H_2_O solvent at pH3 on aluminium foil substrate measured using XPS. Upon removal from solution, films were cured (**solid green squares**) [8], measured using XPS, rinsed with pH3 CH_3_COOH (**open blue squares**) and measured again.

As shown in Figure 6, once PTMS films were cured they appeared to become stable and could not be removed by rinsing; thereby confirming that the curing process forced the condensation reaction to completion, with physisorbed species no longer present on the substrate. While all species were covalently bound once cured, the difference between PTMS films rinsed pre-cure in Figure 5 and post cure in Figure 6 was the potential for water to become trapped under the film during the self-assembly process, reversing the equilibrium reaction. This observation agrees with the mechanism of water displacement presented above whereby an increase in the concentration of water as a by-product of silane condensation shifts the dynamic condensation equilibrium. However, the presence of an oscillatory behaviour in the adsorption profiles of PTMS and PMDMS confirms that the shift in dynamic equilibrium can displace both physisorbed and covalently bound species from the substrate. Failure of the rinsing procedure to remove covalently bound silane species from the substrate in Figure 6 again indicates the presence of a hydrogen bound intermediate and suggests that the difference in substrate coverage observed for non-rinsed PTMS or PMDMS films and their rinsed counterparts was directly related to the extent of physisorbed silane species on the substrate. 

## 4. Conclusions

Through removal of physisorbed species on the substrate of PDMMS films, the rate of conversion from hydrogen bound to covalently bound species during Langmuir self-assembly was inferred as well as confirming the rinse’s effectiveness in removing only those species which were physisorbed species from the substrate. The removal of physisorbed species from the substrate of PMDMS and PTMS revealed the role of both species in the oscillatory growth process. This, along with the stability of the film post cure indicates a shift in the dynamic equilibrium and reversal of the condensation reaction during self-assembly. 

## Supplementary Materials

The Supplementary Materials are available online at https://www.mdpi.com/2073-4360/11/3/410/s1.

## Abbreviations

PTMS	Propyltrimethoxysilane
PMDMS	Propylmethyldimethoxysilane
PDMMS	Propyldimethylmethoxysilane
XPS	X-ray Photoelectron Spectroscopy
