# Supplementary material for: The Role of Physisorption and Chemisorption in the Oscillatory Adsorption of Organosilanes on Aluminium Oxide
Source: Polymers (Basel). 2019 Mar 4;11(3):410. doi: 10.3390/polym11030410 (PMC6473760; doi:10.3390/polym11030410)
Supplement: Supplementary file 1 [file polymers-11-00410-s001.pdf]

Supplementary data

An example of fits for Si 2p and Al2p peaks used in this manuscript is shown in Figure 1 below, the sum of all components was used to calculate a Si:(Si+Al) value of 0.61.

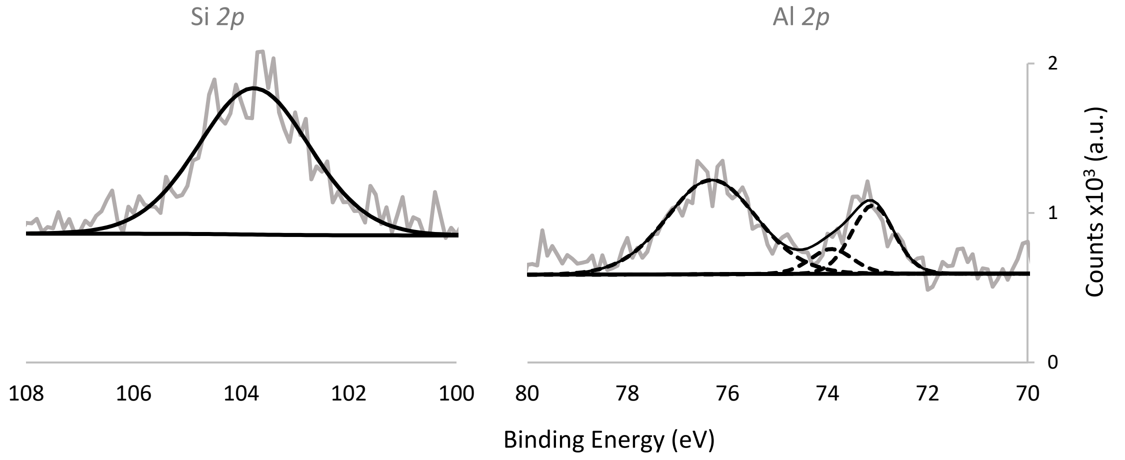

Figure 1: The fit and deconvolution of high-resolution Si 2p (left) and Al 2p (right) XPS spectra of 1% PTMS in aqueous solvent exposed to aluminium oxide for 5 seconds, obtained an Mg Kα X-ray source.
